# Scoping Review of Barriers and Facilitators to Recruitment of Black People With Cancer in Biospecimen-Based Research

**DOI:** 10.1200/PO.23.00708

**Published:** 2024-05-15

**Authors:** Hayley N. Morris, Ashlee Taylor Winslow, Jeenn Alain Barreiro-Rosado, Stacy Torian, Marjory Charlot

**Affiliations:** ^1^ UNC Lineberger Comprehensive Cancer Center, University of North Carolina at Chapel Hill, Chapel Hill, NC; ^2^ University of North Carolina at Chapel Hill School of Medicine, Chapel Hill, NC; ^3^ UNC Gillings School of Global Public Health, University of North Carolina at Chapel Hill, Chapel Hill, NC; ^4^ Division of Libraries, New York University, New York, NY

## Abstract

The increasing focus on precision medicine to optimize cancer treatments and improve cancer outcomes is an opportunity to consider equitable engagement of people racialized as Black or African American (B/AA) in biospecimen-based cancer research. B/AA people have the highest cancer incidence and mortality rates compared with all other racial and ethnic groups in the United States, yet are under-represented in biospecimen-based research. A narrative scoping review was conducted to understand the current literature on barriers, facilitators, and evidence-based strategies associated with the engagement of B/AA people with cancer in biospecimen research. Three comprehensive searches of MEDLINE, CINAHL, Embase, and Scopus were conducted. Of 770 studies generated by the search, 10 met all inclusion criteria for this review. The most frequently reported barriers to engagement of B/AA people in biospecimen research were lack of biospecimen research awareness, fear of medical harm, and violation of personal health information privacy, resource constraints, and medical mistrust. Key facilitators included previous exposure to research, knowledge about underlying genetic causes of cancer, and altruism. Recommended strategies to increase participation of B/AA people in biospecimen-based research included community engagement, transparent communication, workforce diversity, education and training, and research participant incentives. Inclusion of B/AA people in biospecimen-based research has the potential to advance the promise of precision oncology for all patients and reduce racial disparities in cancer outcomes.

## Teaser

This scoping review explores the barriers, facilitators, and strategies for engaging Black individuals with cancer in biospecimen based research.

## INTRODUCTION

The emergence of precision oncology has transformed clinical practice and contributed to improvements in cancer survival and quality of life.^1^ Advancements in precision or personalized cancer medicine have been largely driven by cancer genomic research resulting in the molecular characterization of hematologic and solid cancers and development of therapies directed toward molecular targets.^2^ Human biospecimens (eg, blood, saliva, tissue, urine) have been a critical source for these advancements, yet, in the United States, not all racial and ethnic groups have equitable opportunities to contribute nor to benefit from these advancements.^3^ It is well documented that people racialized as Black or African American (B/AA) in the United States bear a disproportionate burden of poor cancer outcomes, driven largely by structural racism.^4-9^ For most cancers, incidence and mortality rates are highest among the B/AA population compared with all other racial and ethnic groups in the United States.^10^ Under-representation of B/AA people in cancer research likely affects their cancer outcomes given the associated survival gains with clinical trial participation.^11-13^ A recent study on the racial demographics of participants in clinical trials for oncology drugs approved by the Food and Drug Administration demonstrated that the representation of B/AA people was disproportionately low at only 7%.^14^

Representation of B/AA people in biospecimen-based research is also low. Although B/AA people represent 13.6% of the US population,^15^ only 8.3% of biospecimens collected from three of the National Cancer Institute (NCI)–supported Cooperative Human Tissue Network were from B/AA people compared with nearly 90% of those from White Americans (WAs).^16^ In pancreatic cancer Genome Wide Association Studies (GWAS), representation of B/AA people was 3.6% compared with 90% from WAs.^16^ Under-representation of B/AA people in cancer GWAS and other biospecimen-based studies undermines the translation of genomic and molecular data to inform cancer treatment and prevention across racially diverse populations.^17,18^

Efforts to improve diversity and inclusion in biobanking research are necessary and have been evident in the National Institute of Health's Precision Medicine Program *All of Us*. This program has collected biospecimens from approximately one million participants, and as of 2020, 80% of study participants and 51% of those who contributed biospecimens are from minoritized racial and ethnic groups.^19^ These successes have been attributed to engagement of diverse stakeholders, research transparency, and dissemination of research results to study participants.^19^ Similarly, a survey conducted at three NCI-Designated Cancer Centers found that AAs were likely to participate in biospecimen research when community partners assisted with recruitment.^20^ Knowledge and implementation of factors known to promote equitable biospecimen-based research participation to facilitate cancer genomic research are imperative to addressing racial inequities in cancer care and outcomes. We conducted a narrative scoping review to understand the current literature on barriers, facilitators, and evidence-based strategies associated with the engagement of B/AA people with cancer in biospecimen research.

## METHODS

We performed a preliminary search of MEDLINE, the Cochrane Database of Systematic Reviews, and Joanna Briggs Institute (JBI) Evidence Synthesis and found no current or ongoing systematic reviews or scoping reviews focused on barriers, facilitators, and evidence-based strategies for engaging B/AA individuals with cancer in biospecimen-based research. Public health critical race praxis (PHCRP) guided our approach to this review to assess the influence of race (and/or racism) on participation in biospecimen research and engagement of community members in promoting participation of B/AA people in biospecimen research.^21-23^ We used the Preferred Reporting Items for Systematic Reviews and Meta-Analysis (PRISMA) extension for Scoping Reviews criteria and the JBI approach for systematic scoping reviews.^23-25^

### Eligibility Criteria

Inclusion criteria were studies (1) published in the English language in peer-reviewed journals, (2) focused on Black or African American adult populations with cancer in the United States, and (3) that reported barriers and/or facilitators of biospecimen research participation or described strategies for engaging B/AA people in biospecimen research. Exclusion criteria were published literature not meeting the above criteria.

### Information Sources and Literature Search

The supporting librarian (S.T.) developed the search strategy in collaboration with the author (M.C.), drawing on PubMed Medical Subject Headings, the Dictionary of Cancer Terms (NCI), the Glossary of Biospecimen and Biorepository Terms (NCI), terms from the subject matter experts' benchmark articles, and additional terms suggested by health sciences librarian colleagues. On December 8, 2021, S.T. ran the search in Medline (PubMed), Cumulative Index to Nursing and Allied Health Literature, Embase, and Scopus and deduplicated the results in EndNote. Search updates were run on October 12, 2022, and July 25, 2023. Detailed search strategies are included in Appendix Tables A1-A4.

### Study Selection

**FIG 1.**
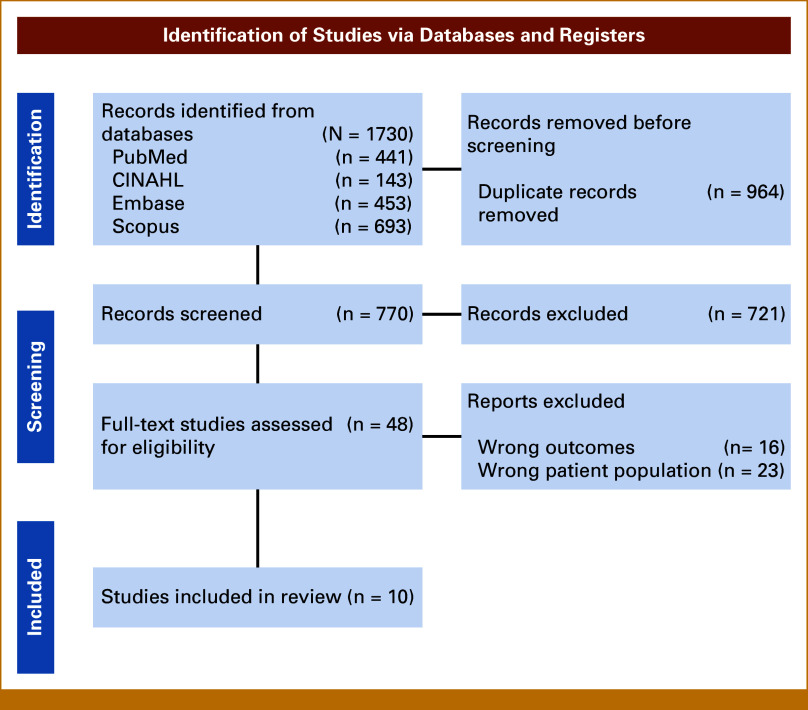
PRISMA diagram.

After duplicates were removed, the search yielded 770 articles. The title and abstract of each article were independently screened by two authors (H.M. and M.C.). Disagreements concerning study eligibility were discussed, and a consensus decision was reached. Screening of abstracts and titles resulted in the exclusion of 721 articles, and an additional 39 articles were excluded during the full-text review. Four authors completed full-text review (H.N.M., J.A.B.-R., A.T.W., M.C.). Reasons for exclusion were wrong patient population (23) and wrong study outcomes (16). Ten articles met all inclusion criteria (Fig 1). Using an a priori extraction tool, data from the 10 articles were categorized according to study characteristics, location, study design, community-engaged approach (yes/no), participants' characteristics, barriers and/or facilitators, recruitment strategies, and study outcomes by three of the authors (H.N.M., J.A.B.-R., and A.T.W.; Table 1).

**TABLE 1.** Study Characteristics (n = 10)

Author, Year of Publication	Location	Study Population (ages, years)	Study Design	Community-Engaged Approach	Recruitment Strategy	Data Collection Methods	Study Objective
Edmonds, 2021^26^	Richmond, VA	Black women with a diagnosis of breast cancer who did not provide a biospecimen during baseline of the WHIP study (21+)	Quantitative, survey-based, cross-sectional	No—this study was a secondary analysis of baseline data from the WHIP prospective study (2012-2017)	From (1) three integrated health networks, (2) teaching hospitals, and (3) community outreach activities (ie, patient identification from patient record screening from community-based oncology practices) Compensation: $15 USD gift card	Tailored letter from PI first sent to participants. Letter included personalized messages about biospecimen donation Assessment survey distributed to measure acceptability of informational materials Biospecimen sample collection kits sent with low-literacy education materials written at a 6th grade reading level that provided instructions on how to provide the sample	Aims were (1) to assess acceptability of brief informational print materials (survey) and (2) to identify demographic and psychosocial factors related to biospecimen donation among women
Ewing, 2015^27^	Howard University (Washington DC)	Black women with a personal diagnosis of, or a family history of, breast cancer, ovarian cancer, or a cancer associated with a hereditary cancer syndrome. (21+)	Secondary analysis: Narrative review comparing effectiveness of three different recruitment strategies used in the Howard University of Genetic Basis of breast cancer Subtype study	Yes—the study discussed in this article (Howard University of Genetic Basis of breast cancer Subtype study) used CBR methods	Study was a discussion of recruitment strategies; no data collection methods described Patient identification by physicians and oncology nursing staff → genetic counselor was notified; tumor registries, churches, tv, and radio advertisements were also used Key representatives from community-based organizations were engaged by the HU CORE group (community Outreach, research and Education) and informed of the active genetic studies and the importance of including Black populations in research; flyers, brochures, presentations, and electronic announcements were created and disseminated throughout the community via respective conduits and partner organizations Phase I: 2-hour consent process, including a 10-page consent form; participants only given the option to provide biospecimens in the form of blood donation Phase II: Phase I + one-on-one or group meeting between physicians and researchers held to discuss benefits of research; African American recruiters were used to help reach target population; engaged community-based organization Phase III: Phase II (except the informed consent process was reduced to 1 hour with a 2-page form); African American GC assisted in recruitment; GCs educated patients about genetic research, genetic testing, and the role of genetics in cancer in the importance of minority participation		To discuss the strategies used to recruit and enroll African American women into the Howard University Genetic Basis of breast cancer Subtype study and present solutions that may dismantle some of the hurdles precluding engagement of minoritized populations in research
Ewing, 2019^28^	Howard University (Washington DC)	Black adults with a medical history of breast, colon, or prostate cancers (21+)	Quantitative, survey-based, cross-sectional	Yes—guided and advised recruitment, study information was distributed to community support groups	Convenience sample was recruited through distribution of study information to community cancer support group members and at cancer center clinic waiting areas at the HUH Cancer Center Snowball sampling was used because HUH serves a predominantly Black population	Self-administered cancer genetics survey Survey was designed to elicit beliefs about genetic research, knowledge about genetics and cancer disparities, and willingness to provide a biospecimen for cancer genetics research	To evaluate the contributions of factors associated with willingness to provide biospecimens for cancer genetic research among African American cancer survivors
Ewing, 2022^29^	Baltimore City and Prince George's county, MD	Unspecified (65-74)	Qualitative focus group-based, cross-sectional	Yes—2 CAGs guided and advised planning (eg, focus group guide development), analysis, and recruitment	Through purposive sampling principles via active outreach efforts facilitated by CAG members or community leaders Compensation: $25 USD Target gift card	FGDs hosted by CAG members at their centers and churches FGDs were about knowledge and previous beliefs related to biospecimen cancer research, community attitudes toward participation, and attitudes toward design features of cancer-related biobanking studies	To better understand older, multiethnic populations' (1) perceptions of the value of cancer biobanking research, (2) study design preferences, and (3) guidance on ways to promote and increase participation
Reddy, 2019^30^	SUNY Downstate medical center (Brooklyn, NY)	Group A: Black adults, cancer status unknown (18-85) Group B: The above criteria + cancer diagnosis	Quantitative, survey-based, cross-sectional	NA	From gastroenterology, primary care and oncology clinics at the SUNY Downstate Medical Center (a large, urban medical center that serves a predominantly African American/Afro-Caribbean population) Participants were also given a 1-page information sheet describing study's purpose	Survey written at fourth to sixth grade reading level, on the basis of the validated biobanking attitudes and knowledge survey Survey also contained questions addressing knowledge and attitudes toward pancreatic cancer section (on the basis of publicly accessible educational materials from the NCI website and from previously published questionnaires assessing colon cancer and prostate cancer in a similar patient population)	To define the psychosocial factors contributing to current disparities in biospecimen collection for research studies among the African American/Afro-Caribbean population
Sheppard, 2018^31^	Georgetown University Medical Center (Washington DC)	Black women with a medical history of breast cancer (21+)	Quantitative, survey-based, cross-sectional	NA	Identified from hospitals via review of health system records and via community outreach (ie, community-based oncology practices) At the clinical sites, research staff sent letters describing the study to consented participants, instructing them to call a number if they wanted to participate or learn more. Other research staff then contacted the women who did not opt out to complete the survey Also via electronic Listserv *v* E-mails that included a link to screening questions that connected interested (if eligible) breast cancer survivors with research staff	Standardized telephone surveys (REDCap) to collect sociodemographic information, sociocultural (ie, religiosity) information, and health care perceptions (ie, medical mistrust, communication with providers, time spent with the doctor); clinical information (ie, stage and treatment type) was abstracted from medical records After baseline survey mouthwash, collection kits were mailed Biospecimen provision was measured by receipt of the saliva kits (yes *v* no)	To (1) assess factors that predict participation in genetic research among breast cancer survivors and (2) fill gaps in knowledge about how sociocultural, health care, and clinical factors affecting women's participation in genetic research in Black and White breast cancer survivors
Li, 2023^24^	Moffitt Cancer Center, Tampa, FL	Adult African American, Black, or Afro-Caribbean prostate cancer survivors	Survey + consent + biospecimen provision via mail	No	First, participants were identified from population-based state cancer registry supported by the Florida Department of Health Then, recruitment consisted of multiple contact waves Wave 1: Potentially eligible individuals were identified via registry and sent an introductory letter describing the study, a study pamphlet, a letter from the cancer registry, a response form with an option to refuse participation, an eligibility screener questionnaire, and a contact information form. Individuals who did not respond were mailed a postcard and called once again to assess eligibility Wave 2: Eligible and interested individuals were sent letter describing study, informed consent form, the study survey, and a contact information form, checklist of materials to be returned to the study team Wave 3: Consented participants were sent a letter describing the remaining study procedures, a saliva collection kit, instructions for sample collection, a medical release form for granting study team's permission to access medical records and prostate cancer tissue specimens, and a contact information form	Participants surveyed about demographics and health behaviors (eg, smoking history, physical activity) + clinical information extracted	To describe the recruitment of African American prostate cancer survivors identified in collaboration with a state-wide cancer registry and rates of consenting to a biobanking study along with sociodemographic and clinical predictors of consent rates
Bishop, 2018^32^	East Alabama Medical Center (Opelika, AL) and all over Alabama	Black adults with breast cancer, ovarian cancer, or prostate cancer Family members who were affected or unaffected with cancer could also joined the study	Secondary analysis: Comparing the effectiveness of 2 recruitment methods: Hospital and community-based	Yes—original Alabama Hereditary Cancer Cohort study included a CBR strategy described in more detail to the right	(Alabama Hereditary Cancer Cohort study): Study participants were identified through both hospital and CBR Hospital (Cancer Center of East Alabama Medical Center): Medical record screening by a research nurse was performed to obtain cancer diagnosis, cancer risk factors, family history, and demographic information Community-based (all over the state of Alabama): Education sessions to cancer support groups, attending Relay for Life events in Alabama counties, participating in other breast cancer–specific community events (ie, walks), and advertisements at community events	(Alabama Hereditary Cancer Cohort study): Medical information was either verbally shared or obtained through the medical record, a family pedigree was generated from this information, and DNA was extracted via a blood sample	To compare the 2 recruitment approaches used in the Alabama Hereditary Cancer Cohort, a study to facilitate the genetic analysis of hereditary breast, prostate, and ovarian cancers. This study targeted underprivileged minority groups living in Alabama
Davis, 2019^25^	Four towns in Louisiana and the LSU Feist-Weller Cancer Center	Black health care providers, primary care and oncology clinic patients (cancer type unspecified) and community members with unknown cancer status	Qualitative, focus group interview-based cross-sectional study	Yes—used in recruitment; conducted focus groups in safety-net primary care and oncology settings and in social, faith-based, and Parkinson's and Alzheimer's caregiver support groups	Patients and community representatives were recruited from the following: A university cancer center An academic safety net medicine clinic An urban and rural Federally Qualified Health Center Two African American sororities An African American church A rural and two urban Council on Aging sites A Volunteers of America Alzheimer's Family Caregiver support group A Parkinson's Caregiver support group Providers were recruited by the medical director at four clinics Compensation: Patients and community group participants were paid $35 USD for their time, and providers were paid $100 USD	2 focus groups (one for patients, the other for providers) that addressed participants' awareness, knowledge, and acceptance of biobanking genomics and clinical trials	(1) to identify factors that may influence the decision of members of under-represented groups to participate in clinical trials and/or biobanking and (2) to elicit their input in crafting clear, culturally appropriate language and recruitment strategies
Drake, 2017^33^	Washington University, in St. Louis School of Medicine, MO	Black men with a history of prostate cancer (40-80)	Qualitative, focus group interview-based, cross-sectional study	Yes—collaboration with the PCCP, an existing group of community members, prostate cancer survivors, and academics who help shape research, education, and outreach opportunities in the region; study included members in both the planning and analysis	Members of the PCCP worked with local community and faith-based groups, fraternal organizations, prostate cancer survivor groups, university-affiliated urology clinics, and health care organizations to identify prospective participants Advertisement was conducted via flyers, and project correspondence was posted in community settings All recruitment material included simple, nonmedical language, and racially matched images of men Focus group venues were community-based and located in places known to have positive relationships with local African American men	Focus groups first addressed participants' pre-existing knowledge, past experience, and general attitude around health research studies The later focus group questions delved more specifically into biospecimen research Participants were finally asked about their general knowledge of biospecimen research, about barriers to participation, particularly for African American men, and about factors that promote participation	To understand barriers and strategies to increase participation in biorepository studies among African American men

Abbreviation: CAGs, community advisory groups; CBR, community-based recruitment; FGDs, Focus group discussions; GC, genetic counselors; HUH, Howard University Hospital; LSU, Louisiana State University; NA, not applicable; PCCP, Prostate Cancer Community Partnership; USD, US dollars; WHIP, Women's Hormonal Initiation and Persistence.

## RESULTS

### Barriers

All 10 studies described barriers to engaging B/AA people with cancer in biospecimen research^24-33^ and were related to lack of awareness, fear, mistrust, logistical concerns, and/or study characteristics (Table 2).

**TABLE 2.** Overview of Barriers to Biospecimen Research

Barriers			
Focus	Theme	Subtheme	Example
Awareness	Lack of knowledge		About biospecimen-based cancer research and its scientific importance^20,24-26,28-30,33^
Logistics	Logistical challenges	Resource constraints and competing demands	Cost of participation,^26,30,33^ limited access to health care settings,^25,31,32^ complex and redundant recruitment methods (eg, multiple recruitment attempts),^24^ lengthy e-mail invitation to participate,^23^ transportation concerns and time constraints^29,33^
Trust/mistrust	Beliefs and fears associated with potential harms	Medical concerns	Concerns for long- and short-term side effects,^29^ fear of pain related to invasive collection procedures,^29,30,33^ fear of uncovering unknown medical condition^33^
		Privacy concerns	Related to security of medical information and biospecimen sample storage^29,30,33^ specifically related to the collaborative nature of biobanks and multiple scientists being engaged^29^; concerns regarding if personal medical information would be used as a barrier to jobs or insurance^25^
	Medical mistrust	Mistrust of researcher intentions	Because of a legacy of racism and historical injustices,^25,31,32^ belief that research will primarily benefit White patients or the institution, not people of color,^25^ regarding placing the study's objectives ahead of participants' well-being^33^
		Lack of research transparency	Regarding how collected biospecimens will be used^25,33^
		Misconceptions about research	Belief that biospecimen research participation negatively interferes with a person's medical care,^29,30^ expectation that participants should be able to reap the immediate benefits of biobanking as opposed to waiting for futuristic promises—therapeutic misconception as the “idea that participation in research yields the same type of information and benefit one would expect to receive when seeking personal medical care,”^29^ belief that pharmaceutical companies may disproportionately benefit and have a conflict of interest^29^
Study/provider characteristics			Providers’ lack time to identify biobanking studies and explain them to patients^25,31^; poor patient-provider communication^31^
Moderators	Characteristics (sociodemographic or clinical) negatively associated with willingness to participate in biospecimen research		Higher self-reported level of religiosity,^26,31^ higher stage of cancer (III or IV compared with 0 or I),^24,26,31^ cancer-associated health conditions or symptoms affecting one's donation ability (ie, lymphedema, dry mouth),^27^ being male,^31^ older age (age 65-79 years *v* age 36-65 years),^24^ receipt of radiotherapy or hormonal therapy to treat their cancer, having government insurance compared with having private insurance,^28^ having no usual place of health care,^28^ patients currently undergoing active treatment (*v* those in the surveillance phase of treatment)^24^

### Awareness

Seven studies identified lack of knowledge about the purpose of biospecimen research as a barrier for participation of B/AA people with cancer in biospecimen research.^25,26,28-30,33^ Lack of patient awareness was often related to lack of understanding of complex research jargon and lack of available research information in the community. Most B/AA individuals in these studies had no knowledge that human blood or other tissues could be used for cancer research and were not familiar with the term “biobanks” as a place of storage for collected biospecimens.^25,29,33^

### Fear

Perceptions of potential harms were cited as barriers to B/AA individuals participating in biospecimen-based research. These included concerns about long- and short-term side effects of specimen donation,^29^ fear of pain related to invasive collection procedures,^29,30,33^ and the fear of uncovering unknown medical conditions.^33^ There was also the belief that biospecimen research participation could negatively interfere with a person's medical care.^29,30^

### Mistrust

Mistrust of medical researcher intentions and lack of transparency of how collected biospecimens would be used were commonly reported barriers.^25,31-33^ One study reported that AAs believed that White populations would disproportionately benefit from medical research.^25^ Another study reported that AAs felt that pharmaceutical companies disproportionately benefited from biospecimen research.^29^ Other studies found that many B/AA participants were not confident that researchers would fairly present research study objectives^33^ nor did they feel that research teams would value their well-being over research outcomes.^31^ There were also concerns related to disclosing personal health information to other medical institutions.^29,33^ Three studies reported that many B/AA patients with cancer did not trust that their personal health information and biospecimen samples would be stored securely.^29,30,33^ One study found that despite being assured of protections, the overwhelming majority of Black patients were still concerned that personal health information would be disclosed, threatening future job opportunities or insurance access.^25^ Several studies indicated that mistrust among B/AA people was largely attributed to the US Public Health Service Syphilis Study at Tuskegee.^25,31,32^

### Logistics

Limited access to health care settings because of difficulty in securing transportation and childcare responsibilities were noted as factors that affected decisions to participate in biospecimen-based research.^25,31,32^ In addition, the financial and opportunity costs of participation, such as taking time off of work with no pay,^26,30,33^ complex and redundant recruitment methods, lengthy enrollment processes,^24^ and the substantial time commitment to engage in research, were barriers for AAs.^29,33^ Additional barriers included having government insurance compared with private insurance,^28^ having no usual place of health care,^28^ and lack of time among clinicians to identify biospecimen studies.^25^

### Patient and Study Characteristics

Seven studies described patient and study characteristics that were negatively associated with willingness to donate biospecimens. Participants' level of religiosity,^26,31^ advanced stage of cancer (stage III/IV compared with stage 0/I),^26,31^ older age (age 65-79 *v* 36-64 years),^24,33^ and cancer-associated symptoms affecting one's ability to donate biospecimens such as lymphedema^27^ were predictors of unwillingness to consent to biospecimen research participation. In addition, patients who were actively receiving treatment (*v* those on surveillance)^24^ were less likely to participate. Studies without monetary incentives were less likely to enroll B/AA participants.^26,30,33^

### Facilitators

Nine studies highlighted facilitators of engaged B/AA people in biospecimen-based cancer research.^25-33^ Facilitators were related to awareness, trust, and altruism (Table 3).

**TABLE 3.** Overview of Facilitators to Biospecimen Research

Facilitators		
Focus	Theme	Example
Awareness	Knowledge	Knowledge about the genetic causes of cancer and the importance of biospecimen-based genetic research^25-28,32,33^
	Exposure to the health care system	Previous participation in medical research,^26,30^ having a personal or family history of cancer^30^
Altruism		Desire to advance medicine,^25,27-30,32^ belief that participation in cancer research will benefit one's family and future generations^25,27-30^
Trust/mistrust	Trust in provider, researcher, or institution	Buy-in/endorsement of a trusted provider,^27,29,31,33^ good reputation of institution^27,33^ on the basis of a family members' or friends' previous experience with a researcher or physician^33^ medical centers/clinics that have well-established and longstanding community engagement and outreach programs^27,33^
	Patient-provider communication	Adequate time with a provider,^31^ physician's positive rapport with the patient base^27,31,33^
Moderators	Characteristics positively associated with willingness to donate biospecimens or likelihood of research participation	Lower stage of cancer (0/I *v* II/IV) compared with higher stages (not metastatic)^24,26,31^; higher perceived well-being^26,31^; financial security^20,28^; being a male cancer survivor (compared with female)^28^; patients undergoing active surveillance (*v* active treatment or no treatment)^24^

### Awareness

Patient knowledge about the genetic causes of cancer and awareness of research opportunities were the most cited facilitators for biospecimen-based research.^20,25-28,32,33^ Previous participation in medical research also improved willingness to participate.^30,31^ One study found that patients with a history of cancer were generally receptive to receiving education about biospecimen research and attending education sessions increased patients' intention to donate biospecimens.^30^

### Trust

Biospecimen research participation among B/AA people was attributed to endorsement from a trusted health care provider.^27,29,31,33^ Specifically, patients were more likely to enroll in a biospecimen-based study when they reported having an established sense of trust in an institution, possibly on the basis of the clinical experiences of friends or family members.^33^ One study demonstrated that the most robust predictor of patient participation in biospecimen or genetic research was satisfaction with the level of provider communication including the length of time patients spent discussing the benefits and risks of research participation with their oncologist.^31^ Similarly, a physician's positive rapport with their patient base^27,31,33^ facilitated biospecimen research participation, as did providers or researchers from programs with well-established community outreach and engagement programs with deeply rooted relationships with community partners.^27,33^

### Altruism

Six studies identified altruism as a facilitator for engagement of B/AA participants in biospecimen-based research. Altruism was characterized as the belief that biospecimen research would potentially help family members and future generation as well as their broader community.^25,27-30,32^ Several studies also found that B/AA participants' desire to advance science facilitated engagement in biospecimen-based research.^25,27-30,32^

### Recruitment and Retention Strategies

All 10 studies discussed strategies to improve the recruitment of B/AA patients with cancer into biospecimen research studies.^24-33^ These strategies included cultural relevance, communication, community engagement, logistical accommodations, and research conduct (Table 4).

**TABLE 4.** Overview of Recruitment Strategies

Evidence-Based Recruitment Strategies		
Focus of Strategy	Theme	Example
Culture	Knowledge	Provision of culturally tailored understandable patient education materials addressing the purpose of biospecimen research^25,26,29-31,33^ and that clarify the difference between research and medical care regarding immediate benefits (dispelling therapeutic misconception),^29^ cultural sensitivity training of health care professionals involved in research recruitment,^29^ education in multiple formats (eg, online, printed)^25,33^ and using simple, everyday language^25,26,29,31,33^
	Cultural concordance of research staff	Research staff (recruiter, physician) are reflective of the community they are serving,^27,29^ having a culturally sensitive, history informed genetic counselor on the research team,^27^ cultural sensitivity training of health care professionals involved in research recruitment,^29^ African American recruiter^27,29^
Logistics	Convenience of participation	Concise informed consent forms,^24,27^ research team traveling to participants to facilitate enrollment,^32^ noninvasive specimen collection procedures,^28,29^ mailed at-home collection kits,^26^ mobile health vans as enrollment stations,^25,32^ research team scheduling a biospecimen procurement procedure (eg, blood draw) at a convenient time (ie, at their physician appointments),^29^ patients given the option to participate using their preferred method (eg, via the Internet, on paper)^24^
	Compensation	Monetary compensation^24,26,30,33^ specifically included incentives in recruitment materials,^24^ provision of childcare support and meal vouchers^33^
Study conduct	Employment of multiple recruitment strategies	Using varied methods of advertisement (ie, education sessions + flyers + social media),^27,33^ + tumor registries,^27^ + physician referrals,^27^ multiple contact waves for recruitment,^24^ postal mail supported by reminders sent via phone or e-mail^24^
	Assurance of confidentiality	Protected health information and biospecimen samples^25,26,28-30^
	Assurance of patient autonomy	Patients have options about the types of biospecimens to donate (ie, saliva or blood),^27,29^ have a say in the use of their samples,^26,27^ duration of specimen storage and study type^29^
Communication and engagement	Engagement of community-based organizations, community members	Community engagement techniques used in recruitment and dissemination,^20,25,27-29,32,33^ usage of community partners to introduce research team to potential participants^20,32^
	Direct physician engagement to promote patient referrals	Physician referrals for recruitment,^27^ meetings between physicians and researchers where researchers explained the translational value of their research in improving health care,^20,27^ optimal time, placement, and presentation of the first invitation to participate^33^
	Patient-provider and patient-biobank communication: follow-up and effective dissemination	Patient receipt of tailored letter from PI including personalized messages about the purpose of biospecimen donation,^26^ patient receipt of study information from medical personnel (in addition to a research recruiter),^33^ follow-up from a biobank that indicates gratitude for the patients' participation,^29^ urgency in returning risk information/research results to community^29^

### Cultural Relevance

Use of culturally representative patient recruitment materials explaining the purpose of biospecimen research^25,26,29-31,33^ in multiple formats (eg, web-based, print)^25,33^ and use of simple, everyday language to describe biobanking were strategies supported by several studies.^25,29,31,33^ Ensuring that research staff, including the study recruiter and physician-researcher, shared the same racial identity as the community from which they were recruiting was another recommended strategy.^27,29^ One study demonstrated that having a Black genetic counselor on the research team serving the role of a health educator and a patient advocate yielded great success in recruiting Black patients for a biospecimen-based research study.^27^

### Communication Strategies

Multimodal communication strategies were used or recommended to improve representation of B/AA participants in biospecimen research studies. Receipt of personalized letters from the primary investigator about the purpose of biospecimen donation^26^ and receipt of study information from clinicians and study coordinators were effective approaches.^33^ Two studies used recruitment flyers or advertisements posted at places of significance for B/AA communities such as churches, hair salons, or broadcasted on B/AA radio stations.^27,32^ Follow-up communication from biobanks or clinics expressing gratitude for research participation and dissemination of research results to the community also engaged B/AA participants in biospecimen-based research.^29^ Phone reminders or other electronic modalities of communication were also used to enhance participation.^24^

### Community Engagement

Seven studies used community-based approaches for recruitment.^20,25,27-29,32,33^ This included community advisory boards to assist with recruitment planning and community members included on the research team. B/AA sororities, cancer survivor support groups, churches, and academic and safety net clinics were also engaged.^27,31,33^ Community partners facilitated introduction of the research team to potential study participants.^20,32^ Academic researchers engaged in discussions with community practice physicians about the translational value of biospecimen-based research, which resulted in increased patient referrals to biospecimen-based studies.^27^ Hospital- and community-based recruitment approaches were compared in a study conducting genetics analyses of hereditary cancer, and 26% of hospital probands compared with 49% of community probands identified as AA.^32^ Greater success of the community-based recruitment approach was attributed to the trust building, educational, and traveling components of that strategy.^32^

### Logistical Accommodations

Nine studies discussed strategies for addressing logistical challenges. Monetary compensation,^24,26,30,33^ childcare services,^33^ meal vouchers,^33^ mitigation of transportation barriers with research teams traveling to community events to recruit potential participants, and use of mobile health vans as enrollment stations were used or recommended.^25,32^ In addition, scheduling biospecimen procurement (eg, blood draw) at the time of a patient's existing physician appointments was recommended.^29^

### Research Conduct

Cancer registries facilitated identification of B/AA participants for a biospecimen-based study given the availability of sociodemographic and clinical data.^24^ Concise informed consent forms was recommended in two studies.^24,27^ Strategies to promote patient autonomy by allowing research participants to determine mode of consent (eg, via Internet, on paper),^24^ type of donation (eg, saliva, blood or tissue),^27,29^ how biospecimens are used,^26,27^ and duration of specimen storage were also suggested.^29^

### Efficacy/Effectiveness of Recruitment Strategies

Only three studies specifically measured outcomes related to effectiveness of interventions to increase the participation of Black people in biospecimen research or consent rates for biobank participation.^24,26,32^ Bishop et al,^32^ demonstrated that community-based recruitment (CBR) resulted in African Americans comprising 70% of people who enrolled in a biospecimen-based research study compared with 26% of those identified through hospital-based recruitment. Edmonds et al^26^ found that receipt of brief informational print materials inclusive of personalized messages increased overall biospecimen donation rate by 17%, and Li et al^24^ demonstrated that consent rates were lowest among prostate cancer survivors who were sent only a lengthy e-mail invitation (2.6%) and highest among those who were sent a prenotification (ie, postcard, postal letter), abbreviated e-mail invitation, and a gift card with the recruitment materials (18%).

## DISCUSSION

To our knowledge, this is the first review to synthesize evidence regarding factors that influence the engagement of B/AA people with cancer in biospecimen research. Strategies that include community engagement, cultural humility training, workforce diversity, incentives for research participation, and transparent communication were recommended to improve participation of B/AA people in cancer-based biospecimen research. These strategies help address barriers attributed to the historical and present-day exploitation and mistreatment of B/AA individuals in research and clinical care and the social determinants of health that disproportionately affect Black people.^34-36^

Engagement of B/AA community leaders from churches, hair salons, and sororities promotes inclusion of B/AA participants in biospecimen-based research and was a strategy used in six studies.^25,27-29,32,33^ Leveraging the lived experiences and perspectives of marginalized communities gives voice to racial inequities in biospecimen-based cancer research and is a core concept of PHCRP.^21,22^ Community engagement builds trust, increases awareness, and promotes participation in biospecimen-based research among B/AA people with cancer. Community engagement has also been successful in increasing awareness and participation of B/AA participants in cancer clinical trials.^37^ Community outreach efforts also improve engagement among people of color without cancer in biospecimen-based research particularly when combined with on-site opportunities to donate specimens.^38^ Use of multipronged community education and outreach efforts are more likely to improve awareness and participation of B/AA individuals in biospecimen-based cancer research.^38-40^

Minimizing research distrust among B/AA communities could be achieved by enhancing cultural knowledge and humility through training opportunities for investigators and research staff.^20^ The importance of clinicians taking the time to understand the needs and history of the communities they served has been highlighted. One study, in particular, identified educating health care professionals about the historically rooted and egregious treatment of B/AA people in medical research and cultural humility training as an opportunity to foster trust.^29^ Workforce diversity also promotes trust. Physician and patient racial concordance is associated with improved cancer research participation among B/AA populations.^41,42^ A recent randomized clinical trial demonstrated that cancer clinical trial information was more trustworthy when delivered by a physician and that racial concordance was specifically associated with trust among Black individuals compared with their White counterparts.^43^

Policies to ensure that research incentives are provided to address social needs or logistical accommodations such as transportation, childcare, and eldercare can mitigate the financial and time cost of biospecimen research participation among B/AA patients with cancer.^24,26,30,33^ In 2018, the ASCO Health Disparities Committee issued a policy statement outlining that recommendations to mitigate financial barriers to cancer clinical trial participation and inclusion of biospecimen-based research should be considered.^44^ The other opportunity to address the social barriers to biospecimen-based research participation is use of patient navigation. In cancer clinical trials, enrollment of Black participants was higher in settings with patient navigators given their expertise in connecting patients to social resources.^45^

Transparent communication regarding how biospecimens are stored, intended use of specimens, and biospecimen research results is an important consideration for potential B/AA research participants and may address negative preconceived notions about research participation.^46-50^ One potential approach to facilitate transparent communication in biospecimen-based research is the use of digital health tools. Dynamic Consent is a patient-facing, mobile application that allows research participants to check the status of their donated biospecimens, withdraw consent, and provide research updates from investigators.^51^ Future studies should consider the cultural relevance of novel digital health tools and assess effectiveness in advancing racial equity in biospecimen-based cancer research.

Although this review provides an important contribution to the literature on factors that influence participation of B/AA people with cancer in biospecimen-based research, there are a few notable limitations. Scoping reviews are exploratory, and therefore, our summary of the literature may not be generalizable. In addition, most of the studies included in this review (seven of 10) were not based on empirical research limiting the ability to assess efficacy or effectiveness of recommended strategies on enrollment of B/AA participants in biospecimen research. Finally, most studies focused on Black adults with breast or prostate cancer, potentially limiting the generalizability of recommended strategies for B/AA adults with other cancers.

Consideration of barriers, facilitators, and strategies to enhance participation of B/AA people with cancer in biospecimen-based research is necessary to further advance precision oncology and help reduce racial disparities in cancer outcomes. Future research should assess the effect of implementing the strategies highlighted in this review on participation of B/AA people in biospecimen-based studies. There is also a need to better understand which multilevel strategies are most effective to address these multifaceted barriers. Qualitative methods should also be considered to provide context on how these strategies support equitable representation of B/AA participants in biospecimen-based research.

## APPENDIX

**TABLE A1.** Database: PubMed

Set No.	Search Strategy
1	("Patient Selection"[Mesh] OR "Patient Participation"[MeSH] OR "Patient Dropouts"[Mesh] OR "Informed Consent"[MeSH] OR "Refusal to Participate"[MeSH] OR "patient selection"[tiab] OR "subject selection"[tiab] OR recruitment[tiab] OR recruits[tiab] OR recruit[tiab] OR recruited[tiab] OR recruiting[tiab] OR retention[tiab] OR retain[tiab] OR retaining[tiab] OR retained[tiab] OR dropout[tiab] OR dropouts[tiab] OR "drop out"[tiab] OR "drop outs"[tiab] OR "dropped out"[tiab] OR participation[tiab] OR participate[tiab] OR participating[tiab] OR participants[tiab] OR participated[tiab] OR represent[tiab] OR represented[tiab] OR representation[tiab] OR underrepresented[tiab] OR underrepresentation[tiab] OR enrollment[tiab] OR enrollment[tiab] OR enroll[tiab] OR enrolled[tiab] OR enrolls[tiab] OR enrolling[tiab] OR consent[tiab])
2	("Black or African American"[Mesh] OR Black[tiab] OR Blacks[tiab] OR "African American"[tiab] OR "African Americans"[tiab] OR Negro[tiab] OR Negroes[tiab])
3	("Biological Specimen Banks"[Mesh] OR "biological specimen bank"[tiab] OR "biological specimen banks"[tiab] OR biofluid[tiab] OR biofluids[tiab] OR biospecimen[tiab] OR biospecimens[tiab] OR bio-specimen[tiab] OR bio-specimens[tiab] OR "biological specimen"[tiab] OR "biological specimens"[tiab] OR biobank[tiab] OR biobanks[tiab] OR bio-bank[tiab] OR bio-banks[tiab] OR biobanking[tiab] OR bio-banking[tiab] OR biorepository[tiab] OR biorepositories[tiab] OR bio-repository[tiab] OR bio-repositories[tiab] OR "tissue research"[tiab] OR "tissue bank"[tiab] OR "tissue banks"[tiab] OR "tumor tissue"[tiab] OR "tumor tissues"[tiab] OR "tissue biopsy"[tiab] OR “tissue biopsies”[tiab] OR "tissue sample"[tiab] OR "tissue samples"[tiab] OR "sample donor"[tiab] OR "sample donors"[tiab] OR "sample donation"[tiab] OR "sample donations"[tiab] OR "sample collection"[tiab] OR "sample collections"[tiab] OR "tissue donor"[tiab] OR "tissue donors"[tiab] OR "tissue donation"[tiab] OR "tissue donations"[tiab] OR "biological sample"[tiab] OR "biological samples"[tiab] OR "collection kit"[tiab] OR "collection kits"[tiab])
4	#1 AND #2 AND #3 AND (English[Filter])

**TABLE A2.** Database: CINAHL

Set No.	Search Strategy
1	MH "Patient Selection" OR MH "Patient Dropouts" OR MH "Consumer Participation" OR MH "Refusal to Participate" OR (MM "Consent (Research)") OR TI ("patient selection" OR "subject selection" OR recruitment OR recruits OR recruit OR recruited OR recruiting OR retention OR retain OR retaining OR retained OR dropout OR dropouts OR "drop out" OR "drop outs" OR "dropped out" OR participation OR participate OR participating OR participants OR participated OR represent OR represented OR representation OR underrepresented OR underrepresentation OR enrollment OR enrollment OR enroll OR enrolled OR enrolls OR enrolling OR consent) OR AB ("patient selection" OR "subject selection" OR recruitment OR recruits OR recruit OR recruited OR recruiting OR retention OR retain OR retaining OR retained OR dropout OR dropouts OR "drop out" OR "drop outs" OR "dropped out" OR participation OR participate OR participating OR participants OR participated OR represent OR represented OR representation OR underrepresented OR underrepresentation OR enrollment OR enrollment OR enroll OR enrolled OR enrolls OR enrolling OR consent)
2	MH "Black Persons" OR TI (Black OR Blacks OR "African American" OR "African Americans" OR Negro OR Negroes) OR AB (Black OR Blacks OR "African American" OR "African Americans" OR Negro OR Negroes)
3	(MH "Tissue Banks+") OR TI ("biological specimen bank" OR "biological specimen banks" OR biofluid OR biofluids OR biospecimen OR biospecimens OR bio-specimen OR bio-specimens OR "biological specimen" OR "biological specimens" OR biobank OR biobanks OR bio-bank OR bio-banks OR biobanking OR bio-banking OR biorepository OR biorepositories OR bio-repository OR bio-repositories OR "tissue research" OR "tissue bank" OR "tissue banks" OR "tumor tissue" OR "tumor tissues" OR "tissue biopsy" OR “tissue biopsies” OR "tissue sample" OR "tissue samples" OR "sample donor" OR "sample donors" OR "sample donation" OR "sample donations" OR "sample collection" OR "sample collections" OR "tissue donor" OR "tissue donors" OR "tissue donation" OR "tissue donations" OR "biological sample" OR "biological samples" OR "collection kit" OR "collection kits") OR AB("biological specimen bank" OR "biological specimen banks" OR biofluid OR biofluids OR biospecimen OR biospecimens OR bio-specimen OR bio-specimens OR "biological specimen" OR "biological specimens" OR biobank OR biobanks OR bio-bank OR bio-banks OR biobanking OR bio-banking OR biorepository OR biorepositories OR bio-repository OR bio-repositories OR "tissue research" OR "tissue bank" OR "tissue banks" OR "tumor tissue" OR "tumor tissues" OR "tissue biopsy" OR “tissue biopsies” OR "tissue sample" OR "tissue samples" OR "sample donor" OR "sample donors" OR "sample donation" OR "sample donations" OR "sample collection" OR "sample collections" OR "tissue donor" OR "tissue donors" OR "tissue donation" OR "tissue donations" OR "biological sample" OR "biological samples" OR "collection kit" OR "collection kits")
4	#1 AND #2 AND #3
5	#4 AND Narrow by Language:- English

**TABLE A3.** Database: Embase (OVID)

Set No.	Search Strategy
1	exp patient selection/
2	exp patient dropout/
3	exp refusal to participate/
4	exp patient participation/
5	exp informed consent/
6	exp patient selection/
7	subject selection.ab,kf,ti
8	recruitment.ab,kf,ti
9	recruits.ab,kf,ti
10	recruit.ab,kf,ti
11	recruited.ab,kf,ti
12	recruiting.ab,kf,ti
13	retention.ab,kf,ti
14	retain.ab,kf,ti
15	retaining.ab,kf,ti
16	retained.ab,kf,ti
17	dropout.ab,kf,ti
18	dropouts.ab,kf,ti
19	drop out.ab,kf,ti
20	drop outs.ab,kf,ti
21	dropped out.ab,kf,ti
22	participation.ab,kf,ti
23	participate.ab,kf,ti
24	participating.ab,kf,ti
25	participants.ab,kf,ti
26	participated.ab,kf,ti
27	represent.ab,kf,ti
28	represented.ab,kf,ti
29	representation.ab,kf,ti
30	underrepresented.ab,kf,ti
31	underrepresentation.ab,kf,ti
32	enrollment.ab,kf,ti
33	enrollment.ab,kf,ti
34	enroll.ab,kf,ti
35	enrolled.ab,kf,ti
36	enrolls.ab,kf,ti
37	enrolling.ab,kf,ti
38	consent.ab,kf,ti
39	1 or 2 or 3 or 4 or 5 or 6 or 7 or 8 or 9 or 10 or 11 or 12 or 13 or 14 or 15 or 16 or 17 or 18 or 19 or 20 or 21 or 22 or 23 or 24 or 25 or 26 or 27 or 28 or 29 or 30 or 31 or 32 or 33 or 34 or 35 or 36 or 37 or 38
40	exp African American/
41	exp Black person/
42	Black.ab,kf,ti
43	Blacks.ab,kf,ti
44	African American.ab,kf,ti
45	African Americans.ab,kf,ti
46	Negro.ab,kf,ti
47	Negroes.ab,kf,ti
48	40 or 41 or 42 or 43 or 44 or 45 or 46 or 47
49	exp biobank/
50	biological specimen bank.ab,kf,ti
51	biological specimen banks.ab,kf,ti
52	biofluid.ab,kf,ti
53	biofluids.ab,kf,ti
54	biospecimen.ab,kf,ti
55	biospecimens.ab,kf,ti
56	bio-specimen.ab,kf,ti
57	bio-specimens.ab,kf,ti
58	biological specimen.ab,kf,ti
59	biological specimens.ab,kf,ti
60	biobank.ab,kf,ti
61	biobanks.ab,kf,ti
62	bio-bank.ab,kf,ti
63	bio-banks.ab,kf,ti
64	biobanking.ab,kf,ti
65	bio-banking.ab,kf,ti
66	biorepository.ab,kf,ti
67	biorepositories.ab,kf,ti
68	bio-repository.ab,kf,ti
69	bio-repositories.ab,kf,ti
70	tissue research.ab,kf,ti
71	tissue bank.ab,kf,ti
72	tissue banks.ab,kf,ti
73	tumor tissue.ab,kf,ti
74	tumor tissues.ab,kf,ti
75	tissue biopsy.ab,kf,ti
76	tissue biopsies.ab,kf,ti
77	tissue sample.ab,kf,ti
78	tissue samples.ab,kf,ti
79	sample donor.ab,kf,ti
80	sample donors.ab,kf,ti
81	sample donation.ab,kf,ti
82	sample donations.ab,kf,ti
83	sample collection.ab,kf,ti
84	sample collections.ab,kf,ti
85	tissue donor.ab,kf,ti
86	tissue donors.ab,kf,ti
87	tissue donation.ab,kf,ti
88	tissue donations.ab,kf,ti
89	biological sample.ab,kf,ti
90	biological samples.ab,kf,ti
91	collection kit.ab,kf,ti
92	collection kits.ab,kf,ti
93	49 or 50 or 51 or 52 or 53 or 54 or 55 or 56 or 57 or 58 or 59 or 60 or 61 or 62 or 63 or 64 or 65 or 66 or 67 or 68 or 69 or 70 or 71 or 72 or 73 or 74 or 75 or 76 or 77 or 78 or 79 or 80 or 81 or 82 or 83 or 84 or 85 or 86 or 87 or 88 or 89 or 90 or 91 or 92
94	39 and 48 and 93
95	article.pt
96	Article in press.pt
97	chapter.pt
98	conference paper.pt
99	conference review.pt
100	erratum.pt
101	review.pt
102	short survey.pt
103	95 or 96 or 97 or 98 or 99 or 100 or 101 or 102
104	94 and 103
105	limit 104 to english language
106	from 105 keep 1-453

**TABLE A4.** Database: SCOPUS

Set No.	Search Strategy
1	TITLE-ABS-KEY("patient selection" OR "subject selection" OR recruitment OR recruits OR recruit OR recruited OR recruiting OR retention OR retain OR retaining OR retained OR dropout OR dropouts OR "drop out" OR "drop outs" OR "dropped out" OR participation OR participate OR participating OR participants OR participated OR represent OR represented OR representation OR underrepresented OR underrepresentation OR enrollment OR enrolment OR enroll OR enrolled OR enrolls OR enrolling OR consent)
2	TITLE-ABS-KEY(Black OR Blacks OR "African American" OR "African Americans" OR Negro OR Negroes)
3	TITLE-ABS-KEY("biological specimen bank" OR "biological specimen banks" OR biofluid OR biofluids OR biospecimen OR biospecimens OR bio-specimen OR bio-specimens OR "biological specimen" OR "biological specimens" OR biobank OR biobanks OR bio-bank OR bio-banks OR biobanking OR bio-banking OR biorepository OR biorepositories OR bio-repository OR bio-repositories OR "tissue research" OR "tissue bank" OR "tissue banks" OR "tumor tissue" OR "tumor tissues" OR "tissue biopsy" OR “tissue biopsies” OR "tissue sample" OR "tissue samples" OR "sample donor" OR "sample donors" OR "sample donation" OR "sample donations" OR "sample collection" OR "sample collections" OR "tissue donor" OR "tissue donors" OR "tissue donation" OR "tissue donations" OR "biological sample" OR "biological samples" OR "collection kit" OR "collection kits")
4	#1 AND #2 AND #3
5	#4 AND (LIMIT-TO (LANGUAGE, "English"))
